# GDF-15 as a Target and Biomarker for Diabetes and Cardiovascular Diseases: A Translational Prospective

**DOI:** 10.1155/2015/490842

**Published:** 2015-07-27

**Authors:** Ramu Adela, Sanjay K. Banerjee

**Affiliations:** Drug Discovery Research Center, Translational Health Science and Technology Institute (THSTI), Faridabad, Haryana 122014, India

## Abstract

Growth differentiation factor-15 (GDF-15) is a stress responsive cytokine. It is highly expressed in cardiomyocytes, adipocytes, macrophages, endothelial cells, and vascular smooth muscle cells in normal and pathological condition. GDF-15 increases during tissue injury and inflammatory states and is associated with cardiometabolic risk. Increased GDF-15 levels are associated with cardiovascular diseases such as hypertrophy, heart failure, atherosclerosis, endothelial dysfunction, obesity, insulin resistance, diabetes, and chronic kidney diseases in diabetes. Increased GDF-15 level is linked with the progression and prognosis of the disease condition. Age, smoking, and environmental factors are other risk factors that may increase GDF-15 level. Most of the scientific studies reported that GDF-15 plays a protective role in different tissues. However, few reports show that the deficiency of GDF-15 is beneficial against vascular injury and inflammation. GDF-15 protects heart, adipose tissue, and endothelial cells by inhibiting JNK (c-Jun N-terminal kinase), Bad (Bcl-2-associated death promoter), and EGFR (epidermal growth factor receptor) and activating Smad, eNOS, PI3K, and AKT signaling pathways. The present review describes the different animal and clinical studies and patent updates of GDF-15 in diabetes and cardiovascular diseases. It is a challenge for the scientific community to use GDF-15 information for patient monitoring, clinical decision-making, and replacement of current treatment strategies for diabetic and cardiovascular diseases.

## 1. Introduction

Prevalence of diabetes is reaching epidemic proportions in young people due to increase in life expectancy, sedentary life style, and obesity. Adults with diabetes and obesity are more prone to cardiovascular complications (World health statistics 2014). As per the International Diabetic Federation (IDF) diabetes atlas (Sixth edition 2013), the number of people with diabetes is 382 million and it is going to rise to 592 million by 2035. Global burden of diabetes is huge and 548 billion dollars was spent in 2013. In India, approximately 65.1 million people are with diabetes [1]. The prevalence, incidence, and mortality of cardio vascular diseases are 2–8-fold higher in persons having diabetes than those without diabetes [2].

Diabetes is characterized by high glucose level in blood due to either less insulin secretion from pancreas or developing insulin resistance in skeletal muscle. Diabetes is categorized into many types; however, two major types of diabetes are type 1 diabetes (T1DM) and type 2 diabetes (T2DM). T1DM is an autoimmune disease and result of autoimmune destruction of *β* cells. Thus patients with T1DM are not able to secrete sufficient insulin in blood or totally lose insulin secretary capacity. T2DM is the commonest form and it is characterized by insulin resistance mostly in skeletal muscle and deficiency of insulin release at end stage. In general, T2DM causes elevation of blood glucose level and other components of metabolic syndrome. Parameters of metabolic syndrome are elevated blood pressure, elevated triglycerides, reduced high density lipoprotein levels, and abdominal obesity. An increase in adipose tissue (abdomen obesity) results in elevation of adipokines, that is, free fatty acids (FFA), tumor necrosis factor (TNF-*α*), C-reactive protein (CRP), interleukin-6 (IL-6), plasminogen activator inhibitor-1 (PAI-1), adiponectin, and leptin. Adipocytokines integrate the endocrine, autocrine, paracrine signals to mediate the insulin sensitivity, oxidative stress, energy metabolism, blood coagulation, and inflammatory responses. Elevated levels of FFA induce insulin resistance and increase fibrinogen and PAI-1. In the long run, high FFA and glucose together impair beta cell function through lipotoxicity and glucotoxicity and develop macro- and microvascular complications [3, 4]. Recently GDF-15 was identified as one of the important plasma markers, which correlates with cardiometabolic syndrome.

Growth differentiation factor-15 (GDF-15) is a member of the transforming growth factor-*β* (TGF-*β*)/bone morphogenetic protein (BMP) super family. GDF-15 is also known as macrophage inhibiting cytokine 1 (MIC-1), placental transformation growth factor (PTGF-*β*), prostate derived factor (PDF), placental bone morphogenetic protein (PLAB), NSAID activated gene-1 (NAG-1), and PL74 [5, 6]. Initially GDF-15 was reported to inhibit TNF-*α* production in lipopolysaccharide-stimulated macrophages and thus named as macrophage inhibitory cytokine-1(MIC-1) [7]. However, Subsequent studies did not confirm the same concept of macrophage suppression [8].

GDF-15 is produced as *a* ≈ 40 kDa propeptide form. The N terminus is cleaved and released as *a* ≈ 30 kDa disulphide linked dimeric active protein form [9]. GDF-15 is a growth factor whose expression increases with age. Biologic age is related to the several markers such as oxidative stress, protein glycation, inflammation, and hormonal changes. Many of these stresses induce GDF-15 expression by either p53 or early growth response protein -1 (EGR-1) transcription factors [10–12]. GDF-15 levels are also affected by environmental factors independently of genetic background. One study found that GDF-15 level is a novel and powerful predictor of all-cause mortality in general population and independent of several markers associated with mortality risk including age, body mass index (BMI), smoking history, IL-6, CRP, and telomere length [13].

**Figure 1:**
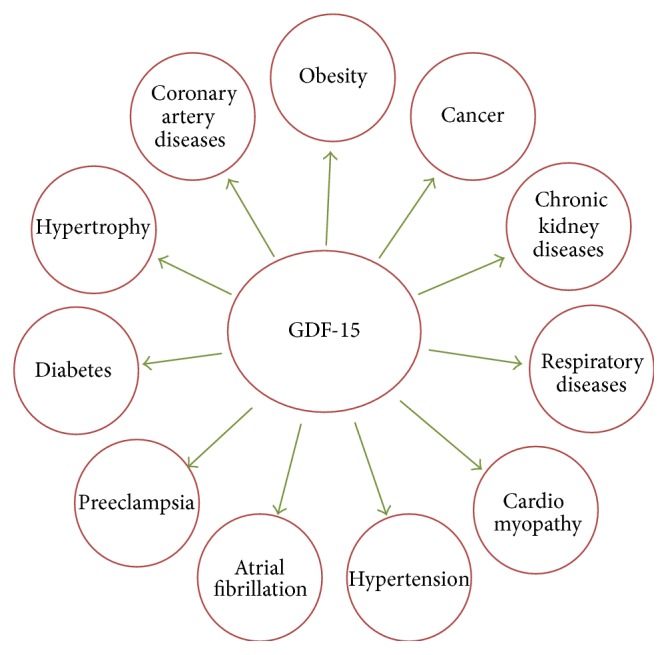
Role of GDF-15 in different diseases conditions. GDF-15 plays an important role to modulate metabolic, cardiovascular, obesity, cancer, and chronic disease.

Higher level of GDF-15 is associated with increased cardiovascular and noncardiovascular mortality; it plays pivotal role in development and progression of cardiovascular diseases such as heart failure, coronary artery diseases, atrial fibrillation, diabetes, cancer, and cognitive impairment (Figure 1) [14, 15]. Increased GDF-15 expression is a feature of many cancers including breast, colon, pancreas, and prostate. Many epithelial tumor cell lines secrete high levels of GDF-15. Several studies showed that higher expression of GDF-15 mRNA and protein was found in cancer biopsies [16–18]. High expression of GDF-15 in tumor is also associated with an increase in serum GDF-15 levels, suggesting the use of serum GDF-15 measurement for the diagnosis and management of cancer [9, 18–20]. In this present review, we described all studies on GDF-15 that reports its role in diabetes and cardiovascular diseases. We explained how GDF-15 could be used as a prognostic and diagnostic biomarker for cardiometabolic diseases. We have also looked into the potential of GDF-15 as a novel target for diabetes and cardiovascular diseases.

## 2. GDF-15 Expression and Release

GDF-15 is highly expressed in the placenta and prostate but also expressed in heart, pancreas, liver, kidney, and colon [6, 46, 47]. It is a stress-induced cytokine and also releases from macrophages [48], vascular smooth muscle cells [49], cardiomyocytes, adipocytes [50], and endothelial cells [51] after tissue injury, anoxia, and proinflammatory cytokines responses. GDF-15 plays a role as an endocrine factor if present in circulation [52]. GDF-15 highly expressed in response to different kinds of cytokines and growth factors like interleukin-1*β* (IL-1*β*), TNF-*α*, angiotensin II, macrophage colony stimulating factor (M-CSF), and TGF-*β*. Tumor suppressor protein p53 also induces GDF-15 and thus acts as a growth inhibitory molecule in tissue [7, 46, 47, 53]. GDF-15 expression is highly induced in cardiomyocytes after ischemia/reperfusion [53]. Increased expression of GDF-15 was observed in the mouse and human heart within hours after myocardial infarction and remains elevated in the infarcted myocardium for several days. Cardiomyocytes in the infarct border zone have been identified as the main source of GDF-15 [54]. There is a controversy regarding the production sites of GDF-15 during heart failure conditions. Although GDF-15 is strongly released from the infracted human heart [54], it may also be released from macrophages [48]. Lok et al. reported that there is no evidence for the myocardial expression of GDF-15 in patients having advanced nonischemic heart failure. However, the circulating GDF-15 levels were increased the same as cardiac troponin and natriuretic peptides levels in serum. This study indicates that GDF-15 may also be released outside of the heart [55, 56]. Strelau et al. reported that GDF-15 is highly expressed in the central nervous system (CNS) and peripheral nervous system (PNS), mainly in the choroid plexus and is secreted into the cerebral spinal fluid (CSF) [57]. Wiklund et al. have stratified the blood GDF-15 levels into three categories, that is, normal (<1200 pg/mL), moderately elevated (1200–1800 pg/mL), and highly elevated (>1800 pg/mL). They reported that 61% people survived when GDF-15 levels were more than 1800 pg/mL^−1^ [13].

## 3. GDF-15, Obesity, and Diabetes

Obesity is a risk factor for diabetes and cardiovascular diseases. Excess body weight is associated with increased health problems and cause increased cardiovascular morbidity and mortality [58]. GDF-15 released from macrophages, liver and white adipose tissue may act as a metabolic regulator. GDF-15 acts as adipokine like adiponectin and leptin [50] and thus has also been termed as cardiokine [59]. Adipokines, in general, regulate the lipid and glucose metabolism, increase insulin sensitivity, regulate food intake and body weight, and protect from chronic inflammation in adipose tissue [3]. Recently Macia et al. found that GDF-15 decreases food intake, body weight, and adiposity and improves glucose tolerance in normal and obesogenic diets [60]. Several human studies dealing with GDF-15 levels in obesity and diabetes are shown in Table 1. Serum GDF-15 levels were increased in obese and type 2 diabetic women and correlated with body mass index (BMI), body fat, glucose, and C-reactive proteins [50]. Vila et al. reported that median interquartile range (IQR) plasma GDF-15 was 427 (344–626) ng/mL in obese patients as compared to the controls 309 (275–411) ng/mL. Increased GDF-15 levels are strongly associated with waist to height ratio, age, arterial blood pressure, triglycerides, creatinine, glucose, insulin, glycated hemoglobin (HbA1c), and C-peptide. Age, insulin resistance, and creatinine were independent predictors of GDF-15 in obese patients [23]. Recently Chrysovergis et al. reported that GDF-15 is a novel therapeutic target in preventing and treating obesity and insulin resistance by modulating metabolic activity through increased expression of key thermogenic and lipolytic genes in brown adipose tissue (BAT) and white adipose tissue (WAT) [61].

**Figure 2:**
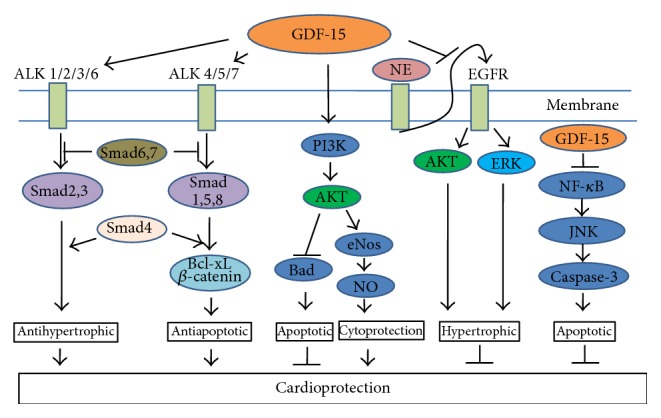
GDF-15 regulates signaling pathways essential for cardioprotection. GDF-15 shows cardioprotective effect through activation of ALK type 1 receptors (ALK 1–7) and phosphorylation of Smad2/3 and Smad1/5/8. After phosphorylation, Smad translocates to the nucleus in the form of heteromeric complex with Smad 4 and activates antihypertrophic pathway. GDF-15 also activates PI3 K/AKT/eNOS/NO pathway and shows cardioprotection. GDF-15 inhibits epidermal growth factor receptor (EGFR) transactivation and NF-*κ*B/JNK/caspase-3 pathway to show its cardioprotective effect.

Hyperglycemia is one of the main chronic symptoms of diabetes. In hyperglycemic conditions, increased reactive oxygen species (ROS) formation leads to cellular injury and cell death [61, 62]. Increased ROS generation in HUVEC cells can cause apoptosis by inhibiting the PI3 K/AKT/eNOS/NO pathway and activation of NF-*κ*B/JNK/caspase-3 pathway [63]. Li et al. proved that increased GDF-15 protects endothelial cells against high glucose induced cellular injury by activating PI3 K/AKT/eNOS signaling pathway and attenuating NF-*κ*B/JNK activation. Nitric oxide production was significantly lower in GDF-15 siRNA transfected HUVEC cells. This study concluded that GDF-15 plays protective role against cell apoptosis through PI3 K/Akt/eNOS pathway but not ERK1/2 and SMAD2/3 (Figure 2) [5]. They found that high glucose increases GDF-15 expression and its secretion, which modulates cell apoptosis in negative feedback manner [5]. As discussed before, GDF-15 is expressed by the adipose tissue through p53, a transcriptional factor that links GDF-15 with obesity and insulin resistance. Expression of GDF-15 is controlled by both p53 dependent and independent mechanisms [46, 64]. Li et al. explained that increase in GDF-15 expression by high glucose in HUVEC cells was p53 dependent pathway. Inhibition of high glucose-induced p53 accumulation by p53 siRNA abolished GDF-15 induction [5]. Obesity promotes p53 activation in adipose tissue and leads to increased production of proinflammatory cytokines, insulin resistance, and diabetes. GDF-15 expression is rapidly induced by proinflammatory cytokines and thus serves as a marker for inflammation in adipose tissue [22, 25, 50, 65]. Insulin resistance and increased GDF-15 both are associated with endothelial dysfunction. The endothelial dysfunction may lead to metabolic derangement, inflammation. and vascular injuries and is associated with increased cardiovascular complications [22]. Increased glucose level in urine and decreased expression of glucose transporters (Glut 1, Glut 2, SGLT 1, and SGLT 2) was observed in type 2 diabetic GDF-15 knockout mice. Similar increased urinary volume was observed in streptozotocin (STZ) induced mice without any alteration of glucose transporters. GDF-15 expression was upregulated within the first 7 days of STZ induced diabetic rats and mice [66]. Khan et al. explained in their study that GDF-15 but not NT-proBNP is raised in patients who have diabetes. Diabetes independently influences the levels of GDF-15 [29]. XENDOS study reported that plasma GDF-15 levels are positively associated with HOMA-IR, an index of insulin resistance [22].

Activated macrophages will secrete proinflammatory, chemotactic cytokines, and chemokines that impair *β* cells function, insulin sensitivity, and infiltration of monocytes into the tissues [24, 67]. Several studies reported that numbers of macrophages were increased in pancreatic islets in type 2 diabetic patients [68]. GDF-15 concentration in plasma was increased in individuals with early stages of T2DM manifestation. Dostalova et al. reported that serum concentration of GDF-15 was increased approximately 1.2- and 2-fold in obese and T2DM women patients, respectively, compared to control subjects. Serum GDF-15 levels are positively correlated with body weight, body fat, triglyceride, glucose, HbA1c, and C-reactive protein [25]. Sugulle et al. observed that plasma GDF-15 levels was elevated in preeclampsia (5978 median (3822–15652 IQR) ng/L) and superimposed preeclampsia in diabetes mellitus (6002 (4230–11830) ng/L) compared to the control subjects (3710 (1860–6266) ng/L) [26]. However some group of scientists believed that it is not associated with the incident type 2 diabetes rather its increase in plasma might be part of anti-inflammatory response for the onset of diabetes [24]. Scientific data showed that serum GDF-15 could be a potential marker to identify individuals who are at risk for diabetes and obesity. However, a longitudinal study should be done where we can identify the early stage of a disease when GDF-15 level starts to increase in serum. More research needs to be carried out to find if administration of GDF-15 has any role to reduce inflammation or early pathological changes in diabetes and obesity.

## 4. GDF-15 and Cardiovascular Diseases

Cardiovascular (CV) diseases, that is, atherosclerosis, hypertension, hypertrophy or heart failure, myocardial infarction (MI), coronary artery disease (CAD), or stroke, are the most prevalent diseases and major cause of the death worldwide [69]. Aging, diabetes, and other risk factors will increase the disease progression by inducing left ventricular hypertrophy, endothelial dysfunction, hypertension, and vascular diseases [65]. GDF-15, the first TGF-*β* protein family, plays a cardioprotective role in the adult heart through activation of Smad2, Smad3, and ALK4/5/7 receptors [70]. GDF-15 is not expressed in heart under normal physiological conditions but increases rapidly in response to cardiovascular injury, such as pressure overload, heart failure, ischemia/reperfusion, and atherosclerosis [54, 71]. Several human studies dealing with GDF-15 levels in cardiovascular diseases are shown in Table 2. GDF-15 showed antiapoptotic effect against ischemia reperfusion (I/R) and reduced the size of myocardial infarction (MI). GDF-15 activates Smad1 and reduces apoptotic cell death through upregulation of Bcl-xL and *β*-catenin. Similarly, BMP-2 also exerts antiapoptotic effect through activation of Smad1. GDF-15 and BMP-2 show similarities in their primary structure and Smad activation. GDF-15 is more close to the BMP-2 family than the TGF-*β* subfamily. BMP-2 activates ALK-2/3/6 and phosphorylates Smad1/5. Similarly, GDF-15 activates type I receptors and Smad1/5 [70]. All these signaling pathways regulated by GDF-15 are responsible for cardioprotection (Figure 2).

GDF-15 predicts adverse outcomes in patients with acute chest pain, MI, or chronic angina [29, 30, 45, 72]. GDF-15 is an emerging biomarker, as it is elevated in early subclinical disease and has prognostic utility for cardiovascular events and mortality [37]. Recent findings showed that GDF-15 levels were associated with lower left ventricular ejection fraction (LVEF), worse diastolic function, greater inducible ischemia, and lower exercise capacity. GDF-15 is also correlated with NT-proBNP, reduced plaque burden, left ventricular mass, concentric left ventricular hypertrophy, coronary artery disease, and heart failure [37, 73]. Role of GDF-15 in different cardiovascular diseases condition is described below.

### 4.1. Hypertrophy

Cardiac hypertrophy is typically characterized by enlargement of the heart associated with an increase in cardiomyocyte cell size in response to physiological stimuli such as exercise and pathophysiological stimuli such as hypertension, ischemic heart diseases, valvular insufficiency, infectious agents, or mutations in sarcomeric genes [71]. Hypertensive patients are more prone to left ventricular hypertrophy (LVH). LVH is an early change for the cardiac damage in hypertension [74]. The prevalence of LVH in hypertensive patients was about 25% to 35% in China [75, 76]. It has been shown that LVH increases the risk of stroke, coronary heart disease, congestive heart failure, arrhythmias, and sudden cardiac death. All these are associated with cardiovascular morbidity and mortality, as all-cause mortality [77, 78]. Serum GDF-15 levels in hypertensive patients were significantly higher than in healthy volunteers and positively correlated with the thickness of the posterior wall of the left ventricle, interventricular septum, and left ventricular mass, as well as the serum level of norepinephrine [79]. Plasma GDF-15 levels in hypertensive patients with LVH was higher than those hypertensive patients without LVH. They observed positive correlation between plasma GDF-15 levels and LVH in hypertensive patients which indicates that GDF-15 may be involved in the development of LVH in hypertension [79]. Hantani et al. found that GDF-15 might be a useful biomarker for discriminating hypertrophic cardiomyopathy (HCM) from hypertensive left ventricular hypertrophy (H-LVH). It was also observed that serum GDF-15 levels were significantly higher in patients with H-LVH than with HCM, and thus GDF-15 is an independent predictor of H-LVH. The data suggest that GDF-15 levels may help to introduce different treatment strategies for treating HCM and H-LVH [80]. One of the recent studies shows that GDF-15 is an autocrine/paracrine factor that attenuates the cardiac hypertrophy in experimental models via SMAD and kinases (PI3 K and ERK) signaling pathways. This study indicates that GDF-15 works through activation of SMAD protein and kinases, that is, PI3 K and ERK mechanism [71].

Intracellular mechanism of TGF-*β* family is divided into Smad dependent and Smad independent pathway. This intracellular mechanism is determined by the type 1 receptors (ALK1 to 7). GDF-15 activates type 1 receptor and phosphorylates Smad2/3 and Smad1/5/8, which translocate to the nucleus in the form of heteromeric complex with Smad4 [70]. Smad4 is a common transcriptional mediator of the Smad dependent pathway. Wang et al. reported that heart specific deletion of* smad4* ^−/−^(*smad4* ^−/−^ mice) showed greater cardiac hypertrophy and heart failure [55]. Xu et al. suggested that Smad dependent pathway can inhibit apoptosis and shows protection against hypertrophy and fibrosis [79]. Xu et al. proposed a protective mechanism of GDF-15 against cardiac hypertrophy and cell death through Smad protein activation. It was explained in the study that GDF-15 shows TGF-*β*/activin-like response through Smad2/3. While Smad2 overexpression showed the similar beneficial effects of GDF-15, overexpression of Smad6 or Smad7 reversed its antihypertrophic effects [78]. GDF-15 treatment transiently activates the Akt and ERK1/2 signaling [71]. Akt activation regulates cardiomyocyte viability [53] whereas ERK1/2 signaling regulates the cell survival [81]. Both pathways are cardioprotective in nature, thus, most of the data confirm that GDF-15 is a novel antihypertrophic as well as cardioprotective regulatory factor [29, 71].

GDF-15 was found to inhibit myocardial hypertrophy through a Smad2/3 pathway in a pressure-induced hypertrophy model [71]. Similarly Xu et al. reported that GDF-15 protects the heart from norepinephrine (NE) induced hypertrophy through Smad independent pathway [71]. Different Smad independent pathways through which GDF-15 works are MAPKs, TAK-1, and PI3 K/AKT pathways [82]. GDF-15 also inhibits norepinephrine-induced myocardial hypertrophy by inhibition of epidermal growth factor receptor (EGFR) transactivation and phosphorylation of downstream kinases, that is, Akt and extracellular signal-regulated kinases (ERK) (Figure 2) [79]. In contrast, GDF-15 results in prohypertrophic effect in cardiomyocytes through a Smad1 pathway [83].

Recently, one study observed that GDF-15 is a novel promising biomarker in heart failure with normal ejection fraction (HFnEF). It is elevated in subjects with either mild or moderate to severe left ventricular diastolic dysfunction (LVDD) regardless of the presence of CAD or other established risk factors frequently associated with HFnNF [84]. Lok et al. reported for the first time that highly elevated GDF-15 levels can be reversible in some extent, after measuring GDF-15 before and after intervention with left ventricular assist device (LVAD) in New York Heart Association (NYHA) class IV, nonischaemic, and nonvalvular HF patients. They found that the GDF-15 levels were gradually reduced after implantation. This finding suggests that GDF-15 could be used as prognostic marker to measure the response to a potentially life-saving therapeutic intervention such as LVAD implantation [56]. Other studies like valsartan heart failure trial (Val-HeFT trial) indicated that higher GDF-15 levels are associated with many pathological processes and then linked to the severity and progression of heart failure (HF), including neurohormonal activation, inflammation, myocyte death, and renal dysfunctions. Higher GDF-15 levels are associated with adverse outcomes independent of established clinical and biochemical risk factors [40, 65]. Recently, Chen et al. demonstrated that olmesartan prevents cardiac rupture in mice with myocardial infarction (MI) through inhibition of apoptosis and inflammation and is associated with downregulation of p53 activity and upregulation of myocardial GDF-15 [85]. In contrast, irbesartan (AT1 receptor blocker) significantly reduced angiotensin II induced GDF-15 expression in cardiomyocytes [86]. The above studies indicate that angiotensin receptor blockers regulate GDF-15 expression. However, more therapeutic intervention studies are needed to understand whether GDF-15 can be used as a prognostic marker for therapeutic intervention for different cardiovascular disorders.

### 4.2. Atherosclerosis

Development and progression of atherosclerotic plaques are driven by endothelial dysfunction, oxidized low-density lipoprotein (oxLDL) deposition in the subendothelial space, recruitment of inflammatory monocytes to the arterial vessel wall, their differentiation into activated macrophages, and subsequent transformation into cholesterol-laden foam cells in the subendothelial space [87]. It has been shown that GDF-15 inhibits proliferation of endothelial cells (ECs)* in vitro* and* in vivo*. A recent study demonstrated that GDF-15 at high concentration (50 ng/mL) inhibits EC proliferation, whereas, at lower concentrations (5 ng/mL), GDF-15 caused endothelial cell proliferation and was found proangiogenic [50, 88–90].

Transforming growth factor betas (TGF*β*s) have been involved in many of the pathophysiological process mainly in the vascular diseases. It will act as inflammatory markers in advanced stage of atherosclerosis and play a role in pathogenesis of ischemic heart diseases. TGF*β*s involved in the pathogenesis of atherosclerosis by activating proteolytic mechanism of activated macrophages [91–94]. These activated macrophages will undergo apoptosis in lipid rich plaque condition. This phenomenon pointing out that lipid content and inflammatory cell viability may be responsible for the thrombogenicity [95]. Activation of caspase 3, induction of manganese superoxide dismutase (MnSOD), and increase in expression of p53 were seen in human atherosclerotic plaques [96–100]. Signal transduction of oxLDL and its mediator's ceramide and TNF-*α* induce apoptosis in human activated macrophages [97, 98, 101–103]. Poly(ADP-ribose) polymerase (PARP), c-Jun-AP-1, and apoptosis inducing factor (AIF) were detected in apoptotic cells [49, 97–101, 103]. Recently, Schlittenhardt et al. found that GDF-15 is expressed in macrophages after stimulation by several biological mediators, including tumor necrosis factor *α* (TNF-*α*), C6-ceramide, interleukin-1 (IL-1), macrophage-colony stimulating factor (M-CSF), oxLDL, and hydrogen peroxide. In human atherosclerotic carotid arteries, GDF-15 (immunoreactivity) was exclusively localized in activated macrophages and colocalized with oxLDL, MnSOD, AIF, caspase-3 (CPP32), PARP, c-Jun/AP-1, and p53. GDF-15 is supposed to contribute to modulation of apoptosis and inflammatory processes of activated macrophages. All data suggested that increased expression of GDF-15 is associated with the development and progression of atherosclerotic plaques, possibly through the regulation of apoptotic processes [49]. In support of previous statement another study demonstrated that GDF-15 deficiency attenuates early atherogenesis and improves plaque stability by attenuating CCR2 (C-C chemokine receptor type 2) mediated macrophage chemotaxis. Similarly, deficiency of GDF-15 in leukocytes improves atherosclerotic plaque stability by impairing macrophage migration and inducing collagen deposition. A novel function of GDF-15 is to regulate the CCR2-dependent macrophage chemotaxis and proceeds via TGF-*β* receptor II and its downstream effector GRK-2 [104].

Another function of GDF-15 is to control inflammatory process in cells. GDF-15 deficiency attenuates atherosclerosis by regulating interleukin-6 dependent inflammatory response to vascular injury. GDF-15 deficiency results in inhibition of atherosclerosis in mice despite an inhibition of apoptotic processes and an increase in cell density in atherosclerotic lesions. This implicates that inhibition of apoptosis acts as antiatherogenic. Regulation of apoptosis through GDF-15 may be a therapeutic strategy to control atherosclerosis and plaque progression [87]. Triglyceride-rich lipoproteins upregulate GDF-15 by >5-fold in human smooth muscle cells of coronary arteries [48]. This increased GDF-15 may trigger the prognosis of the diseases. Overall GDF-15 levels are increased in cardiovascular disease patients [105] and enhance the risk of atherosclerosis. However, GDF-15 deficiency in leukocytes protects against atherosclerosis [104].

### 4.3. Coronary Artery Diseases and Myocardial Infarction

Coronary artery disease (CAD) is a chronic degenerative condition. CAD is a combination of different clinical syndromes including stable angina, acute coronary syndrome (ACS), heart failure, arrhythmia, and death. Myocardial infarction, a condition associated with coronary artery disease, contributes to deaths [106]. Circulated levels of GDF-15 levels are increased in patients who are admitted to the hospital with an acute coronary syndrome. This was proved in “non-ST segment elevation ACS” patients in GUSTO 4 trial [106]. People with elevated levels of GDF-15 (>1800 ng/L) had a high risk for mortality within one year [105]. However, increased GDF-15 level has beneficial role during invasive strategy. In the “Fast Revascularization during in Stability in Coronary artery disease II” (FRISC-II) trial with GDF-15 level <1200 ng/L did not show any benefit from the invasive strategy even though they had ST segment depression or a troponin T level >0.01 *μ*g/L. Patients with GDF-15 levels >1200 ng/L, especially those with 1800 ng/L experienced significant reduction in the combined end point of death or myocardial infarction by the routine invasive strategy [32].

A recent Dallas Heart Study suggests that higher GDF-15 is associated with prevalent coronary artery calcium (CAC) and cardiovascular mortality. People having GDF-15 concentrations ≥1800 ng/L were at increased risk for all-cause and cardiovascular death compared to those with <1200 ng/L. Increasing GDF-15 levels were associated with increasing age, diabetes, renal dysfunction, and inflammatory marker (CRP). Increasing GDF-15 was significantly correlated with black race, smoking, and hypertension. Increasing NT-proBNP concentrations are less associated with increasing GDF-15 levels, but there were no associations with BMI and sex differences [33].

Recently, elevated circulating GDF-15 levels that measured in individuals with acute myocardial infarction have been correlated with inflammatory biomarkers, suggesting a link between GDF-15 and inflammation [30, 32]. After cardiac surgery acute muscle wasting will occur because of imbalance between muscle atrophy and hypertrophy. Bloch et al. found that GDF-15 may be responsible for the skeletal muscle wasting in humans [31].

Recent study demonstrated that GDF-15 protects against fatal cardiac rupture in a mouse model of myocardial infarction. Induction of GDF-15 locally in the infarcted heart reduces the cardiac rupture by acting as an anti-inflammatory cytokine and represses myeloid cell recruitment into the infarcted area [107]. GDF-15 also inhibits chemokine-triggered *β*2 integrin activation on myeloid cells by activating Cdc42 and inhibition of small GTPase Rap1. Thus, GDF-15 is an inhibitor of leukocyte integrin that is one of the essential components to induce cellular injury after myocardial infarction [107].

In summary, GDF-15 is a very promising diagnostic marker for mild to moderate heart failure with normal ejection fraction or the absence of coronary artery diseases. However, more studies need to be done to distinguish different kinds of heart failure using GDF-15. Higher level of GDF-15 can predict the mortality for CAD patients. Some studies also showed its potential to use GDF-15 as a prognostic marker for therapeutic intervention for different cardiovascular disorders. Although the use of GDF-15 as a biomarker for cardiovascular disease is well established, its therapeutic application is debatable. While GDF-15 can show protection against cardiac hypertrophy, its increased expression is associated with the development and progression of atherosclerotic plaques. Further research is essential before considering GDF-15 as therapeutic intervention against cardiovascular diseases.

## 5. GDF-15 and Kidney Disease: Linkage between Diabetes and Cardiovascular Diseases

Diabetic nephropathy is a progressive kidney disease and a well-known complication of long standing diabetes [108]. Myocardial damage is directly associated with the development of proteinuria and focal glomerulosclerosis [109]. Increase in circulating troponins was observed commonly in patients with chronic kidney diseases (CKD) and are associated with the kidney disease progression and death. The association of circulating troponin and kidney damage is related to cardiac injury, rather than diminished clearance [110]. Higher plasma GDF-15 was associated with incident of CKD and indicates rapid decline in renal function [111]. Higher levels of GDF-15 were predictive of deterioration of kidney function [21]. Studies on renal injury in animal models suggest two possible reasons for increase in GDF-15 levels during renal diseases; either GDF-15 is less cleared from the circulation by the kidneys or synthesis of GDF-15 is increased in renal diseases, or both [112, 113]. In diabetic renal injury, increases in urinary GDF-15 were associated with proximal tubule injury [114]. Thus the hypothesis of less clearance of GDF-15 from kidney is not true. Renal GDF-15 expression also appears to be upregulated in response to metabolic acidosis [113] and kidney injury [113]. van Huyen et al. found that higher levels of GDF-15 are a predictive marker of cardiovascular mortality in patients with diabetic nephropathy besides other well-known cardiovascular risk factors like NT-proBNP and glomerular filtration rate (GFR) [113]. Plasma GDF-15 levels were also increased with the mogensen stage in type 2 diabetic nephropathy, and, thus, it is an independent risk factor for increased microalbuminuria (mAlb). It is significantly correlated with mAlb and eGFR, and thus GDF-15 would be useful in early diagnosis, evaluation, and prediction of the outcomes of type 2 diabetic nephropathy [28]. Although some studies have been performed to find the correlation of plasma and urine GDF-15 levels with kidney disease, there is no study to look into its potential as prognostic marker for kidney disease after intervention.

## 6. GDF-15 and Nitric Oxide: Cross Talk in Diabetes and Cardiovascular Diseases

Increased levels of GDF-15 were associated with reduced endothelium-dependent vasodilation in resistance vessels [73]. GDF-15 shows protective effect against high glucose induced endothelial cell injury by activation of PI3 K/AKT/eNOS signaling pathway. GDF-15 is important to release NO level in endothelial cells. In a recent study, nitric oxide production was significantly lower in GDF-15 siRNA transfected HUVEC cells. On the other hand NO is also responsible to increase GDF-15 gene expression [5]. Nitric oxide is responsible to alter the gene expression through cGMP dependent and cGMP independent signaling pathway. In cGMP independent pathway, nitric oxide reacts with superoxide to form peroxynitrate. Kempf et al. observed that nitric oxide increased GDF-15 expression in cardiomyocytes through superoxide/peroxynitrate dependent pathway, a c-GMP independent pathway [54]. Endothelial dysfunction or injury due to diabetes and smoking may induce inflammation and generate oxidative stress within the vessel wall [115]. Several authors mentioned that oxidative stress and proinflammatory cytokines can induce GDF-15 expression in macrophages and different other cells. Thus increased GDF-15 is linked to oxidative stress, inflammation, and endothelial dysfunction [34, 49, 73, 116–118]. Further research is still needed to understand whether GDF-15 can modulate NO levels or vice-versa in other nonendothelial cells.

## 7. GDF-15 a Potential Biomarker

In the last two decades, we have enormous improvement in the biomarker discovery but only few biomarkers gained wide spread use in clinical practice such as troponin T, troponin I, Nt-proBNP, and B-type natriuretic peptide (BNP) [14]. Recently, Kahli reported that GDF-15 levels increased gradually during and after coronary bypass grafting. This study concluded that GDF-15 levels might be used as a marker of cardiac injury and renal dysfunction [42]. Zhang et al. performed a study to find out multimarkers as predictors of cardiovascular events in patients with mild to moderate coronary artery lesions. This study examined nine plasma inflammatory cytokines, that is, cathepsin S, chemokine (C-X-C motif) ligand 16 (CXCL16), sopluble CD40 ligand, IL-10, placental growth factor, GDF-15, MMP-9, monocyte chemo attractant protein-1, and hs-CRP in 964 patients having mild to moderate lesions, and assessed their association with risk of cardiovascular events during 3 years of their follow-up study. It was concluded that cathepsin S, sopluble CD40 ligand, placental growth factor, and GDF-15 were instructive biomarkers for predicting cardiovascular diseases. This study showed that multimarkers approach is useful to significantly predict cardiovascular diseases progression than the individual marker approach [119].

Similarly, Schnabel et al. investigated 12 biomarkers including GDF-15, related to inflammation, lipid metabolism, renal function, and cardiovascular function and remodeling. These markers are C-reactive protein, GDF-15, neopterin, apolipoproteins AI, B100, cystatin C, serum creatinine, copeptin, C-terminal-proendothelin-1, midregional-proadrenomedullin (MR-proADM), midregional-proatrial natriuretic peptide (MR-proANP), and N-terminal-pro-B-type natriuretic peptide (Nt-proBNP). Blood was collected from 1781 stable angina patients in relation to nonfatal myocardial infarction and cardiovascular death (*n* = 137). The study concluded that Nt-proBNP, GDF-15, MR-proANP, cystatin C, and MR-proADM are the strongest predictors of cardiovascular outcome among patients with stable angina [120].

Similar to other biomarkers, GDF-15 can also be used for diagnosis of diseases and help to select the therapy. GDF-15 diagnosis method is patented for diagnosing any subject suffering from an acute inflammation. GDF-15 is also patented for the diagnosis of kidney injury after surgery, prediction of kidney failure after heart surgery, and detection the prognosis of chronic kidney diseases. GDF-15 is patented as a biomarker for the type 1 diabetes and diabetes related heart diseases. Besides using GDF-15 as a biomarker, GDF-15 polypeptide itself is patented to treat or ameliorate metabolic disorders.  Table 3 described briefly the list of patents with GDF-15 that used as biomarker for diabetes, cardiovascular disease, and kidney disease.

## 8. Conclusion

Previous studies revealed that GDF-15 could be a prognostic and diagnostic marker for the cardiovascular and diabetic diseases. Proper reference ranges of GDF-15 need to be established to identify the disease severity and risk stratification of the diseases. However, before accepting as a clinically useful biomarker, the following questions need to be answered. (1) Whether GDF-15 measurement can support therapeutic management? (2) Can it be used for the routine clinical practice or clinical measurement? (3) Whether GDF-15 level can give any diagnostic and prognostic information? (4) Whether it can be used to take clinical decision for any particular diseases like B-type natriuretic peptide (BNP) for the heart failure and troponin for the acute coronary syndrome (ACS). (5) GDF-15 can be used as a single marker or multi marker approach along with other individual marker. There is very little information regarding pathophysiological role of GDF-15 in diabetes, CAD, hypertension, and diabetes associated with cardiovascular diseases. More intervention studies like AT1 receptor antagonist need to be carried out to bring GDF-15 as a prognostic marker for diabetic and cardiovascular diseases. Further understanding regarding the signaling pathways of GDF-15 may help to discover novel therapies against diabetes and cardiovascular complications.

## Figures and Tables

**Table 1:** List of human studies dealing with GDF-15 levels in obesity and diabetes.

Disease/population/follow-up period	Sample size	Major findings	Reference
T1DM patients with diabetic nephropathy (8.1 years)	451	GDF-15 can be used to detect faster deterioration of kidney function	[21]

Obese nondiabetic (XENDOS) trial (4 years follow Up period)	496	GDF-15 is altered among patients having abdominal obesity and insulin resistance and independently associated with future insulin resistance and abnormal glucose control	[22]

Morbidly obese patients	118	GDF-15 changes following bariatric surgery suggest an indirect relationship between GDF-15 and insulin resistance	[23]

Type 2 diabetes (whitehall II study) (11.5 ± 3.0 years follow up period)	552	Baseline GDF-15 concentrations were increased in individuals before type 2 diabetes manifestation	[24]

Patients with obesity and/or obesity and type 2 diabetes mellitus	54	Elevated GDF-15 levels in patients with obesity are further increased by the presence of T2DM	[25]

Preeclampsia and diabetic pregnancies	267	GDF-15 is dysregulated, both in preeclampsia and in diabetic pregnancies	[26]

Patients with T2DM included in screened for the presence of diabetic cardiomyopathy (DC)	T2DM (*n* = 213)	GDF-15 represents a useful and novel tool to screen diabetic cardiomyopathy (DC) in patients with type 2 DM	[27]

Diabetic nephropathy	T2DM (*n* = 30), microalbuminuria (*n* = 20), macroalbuminuria (*n* = 30) patients	Suggesting its value in early diagnosis, evaluation, and prediction of the outcomes of type 2 diabetic nephropathy	[28]

**Table 2:** List of human studies dealing with GDF-15 levels in cardiovascular diseases.

Disease/population/follow-up period	Sample size	Major findings	Reference
Acute myocardial infarction [AMI]	1142	GDF-15 is a prognostic marker of death and HF in patients with AMI Multimarker approach with GDF-15 and NT-pro-BNP is more informative than either marker alone and may be useful for risk stratification in AMI patients	[29]

Acute coronary syndrome [ACS] (PROVE IT-TIMI 22)	3501	GDF-15 is altered with recurrent events after ACS. GDF-15 may be used as a prognostic marker in ACS	[30]

Human model of acute muscle wasting following cardiac surgery	42	GDF-15 is a potential novel factor associated with muscle atrophy, which may become a therapeutic target in patients with ICU acquired paresis and other forms of acute muscle wasting	[31]

Non–ST-elevation ACS (FRISC-II) trial (2 years)	2079	GDF-15 is a potential tool for risk stratification and therapeutic decision making in patients with non-ST-elevation acute coronary syndrome	[32]

General adult population (Dallas Heart Study) (7.3 years follow up period)	3219	GDF-15 is independently marker for subclinical coronary atherosclerosis and mortality	[33]

Framingham Offspring cohort participants (9.5 years follow up period)	2614	Higher circulating GDF-15 was observed with incident renal outcomes and improves risk prediction of incident chronic kidney diseases (CKD)	[34]

Hypertensive left ventricular hypertrophy (H-LVH), hypertensive cardiomyopathy (HCM)	149	GDF-15 might be a useful biomarker for discriminating HCM from H-LVH	[35]

Patients with preclinical diastolic dysfunction or heart failure with normal ejection fraction (HFnEF)	119	GDF-15 levels are elevated in subjects with HFnEF and can differentiate normal diastolic function from asymptomatic LVDD	[36]

Patients with stable ischemic heart disease (Heart and Soul study) (8.9 yrs follow-up period)	984	Higher GDF-15 level was observed with major cardiovascular (CV) events in patients with stable ischemic heart disease	[37]

Untreated hypertensive patients	299	Plasma GDF-15 level was increased with LVH in hypertensive patients	[38]

71-year-old men (ULSAM study)	940	In elderly men, GDF-15 improves progression of both cardiovascular, cancer mortality, and morbidity beyond established risk factors and biomarkers of cardiac, renal dysfunction, and inflammation	[39]

Heart failure (Val-HeFT study)	1734	Providing independent prognostic information in heart failure	[40]

Coronary artery diseases (CAD)	CAD (*n* = 348) and (*n* = 205) controls	Significant differences of GDF-15, IMA, and PAPP-A in patients with CAD. GDF-15 might be associated with severity of CAD	[41]

Coronary Artery Bypass Grafting with Cardiopulmonary Bypass	34 patients	GDF-15 levels were increased substantially and it is associated with the renal and cardiac biomarkers	[42]

Patients on maintenance hemodialysis	Hemodialysis (*n* = 87), and controls (*n* = 45)	Relation between GDF-15, mortality, and carotid artery thickening suggests that GDF-15 may be a novel marker of atherosclerosis, inflammation, and malnutrition in hemodialysis patients	[43]

ST segment elevation myocardial infarction (STEMI) (3 years)	Patients with STEMI (*n* = 216)	High GDF-15 level is a strong predictor of death and heart failure in patients with STEMI. Although patients with higher GDF-15 levels tend to have lower LV ejection fraction	[44]

Acute chest pain (APACE study)	646	GDF-15 is a better predictor of mortality than of nonfatal CV events	[45]

**Table 3:** GDF-15 patents related to diabetes, cardiovascular diseases, and chronic kidney diseases.

Patent	Applicant	Title
WO2011144571A2	F. Hoffmann-La Roche Ag	GDF-15 based means and methods for survival and recovery prediction in acute inflammation

WO2012138919A2	Amgen Inc.	Method of treating or ameliorating metabolic disorders using GDF-15

WO2012146645A1	F. Hoffmann-La Roche Ag	Diagnosis of kidney injury after surgery

EP2336784A1	Roche Diagnostics GmbH	GDF-15 and/or Troponin T for predicting kidney failure in heart surgery patients

EP2388594A1	Roche Diagnostics GmbH	GDF-15 based means and methods for survival and recovery prediction in acute inflammation

WO2009141357A1	Roche Diagnostics Gmbh, F. Hoffmann-La Roche Ag	GDF-15 as biomarker in type 1 diabetes

US 8,771,961 B2	Roche Diagnostics Operations, Inc.	Monitoring myocardial infarction and its treatment

EP1884777A1	Medizinische Hochschule Hannover	Means and methods for assessing the risk of cardiac interventions based on GDF-15

EP2439535A1	F. Hoffmann-La Roche AG	Diagnosis of diabetes related heart disease, GDF-15 and Troponin as predictors for the development of type 2 diabetes mellitus

WO2013113008A1	Amgen Inc.	GDF-15 polypeptides-ameliorating metabolic disorders

WO2010048670A1	St. Vincent's Hospital Sydney Limited	Method of prognosis in chronic kidney disease

WO2011073382A1	Roche Diagnostics Gmbh	GDF-15 and/or troponin T for predicting kidney failure in heart surgery patients

## Abbreviations

ACS: Acute coronary syndrome
AKT: Serine/threonine kinase (protein kinase B)
AIF: Apoptosis inducing factor
AP-1: Activator protein-1
APACE: Acute coronary syndrome evaluation
Bcl-xL: B-cell lymphoma-extra large
BMI: Body mass index
BMP-2: Bone morphogenetic protein-2
BNP: B-type natriuretic peptide
CAC: Coronary artery calcium
CAD: Coronary artery diseases
Cdc42: Cell division control protein 42
CKD: Chronic kidney diseases
CCR2: C-C chemokine receptor type 2
cGMP: Cyclic guanosine monophosphate
CXCL16: Chemokine (C-X-C motif) ligand 16
EGR-1: Early growth response protein-1
ERK: Extracellular signal-regulated kinases
eNOS: Endothelial nitric oxide synthase
GRK-2: G protein-coupled receptor kinase 2
HbA1c: Glycated hemoglobin
H-LVH: Hypertensive left ventricular hypertrophy
hs-CRP: High-sensitivity C-reactive protein
HUVEC: Human vascular endothelial cells
ICU: Intensive care unit
IDF: International diabetic federation
IL-10: Interleukin-10
IMA: Ischemia modified albumin
JNK: Jun-N-terminal kinase
LVAD: Left ventricular assist device
LVH: Left ventricular hypertrophy
mAlb: Microalbuminuria
MMP-9: Matrix metalloproteinase 9
MR-proADM: Midregional-proadrenomedullin
MR-proANP: Midregional-proatrial natriuretic peptide
NF-*κ*B: Nuclear factor kappa B
Nt-proBNP: N-terminal-pro-B-type natriuretic peptide
oxLDL: Oxidized low-density lipoprotein
PI3 K: Phosphoinositide 3-kinase
PARP: Poly (ADP-ribose) polymerase
PAI-1: Plasminogen activator inhibitor-1
PAPP-A: Pregnancy associated plasma protein-A
T1DM: Type 1 diabetes mellitus
T2DM: Type 2 diabetes mellitus.
